# Successful wayfinding in age: A scoping review on spatial navigation training in healthy older adults

**DOI:** 10.3389/fpsyg.2022.867987

**Published:** 2022-08-16

**Authors:** Madeleine Fricke, Christina Morawietz, Anna Wunderlich, Thomas Muehlbauer, Carl-Philipp Jansen, Klaus Gramann, Bettina Wollesen

**Affiliations:** ^1^Department of Biological Psychology and Neuroergonomics, Technical University of Berlin, Berlin, Germany; ^2^Division of Movement and Training Sciences/Biomechanics of Sport, University of Duisburg-Essen, Essen, Germany; ^3^Institute of Sports and Sports Sciences, Heidelberg University, Heidelberg, Germany; ^4^Clinic for Geriatric Rehabilitation, Robert-Bosch-Krankenhaus Stuttgart, Stuttgart, Germany; ^5^Department of Human Movement Science, University of Hamburg, Hamburg, Germany

**Keywords:** healthy older adults, cognitive aging, spatial orientation, wayfinding, spatial navigation

## Abstract

**Introduction:**

Spatial navigation is a complex cognitive function that declines in older age. Finding one’s way around in familiar and new environments is crucial to live and function independently. However, the current literature illustrates the efficacy of spatial navigation interventions in rehabilitative contexts such as pathological aging and traumatic injury, but an overview of existing training studies for healthy older adults is missing. This scoping review aims to identify current evidence on existing spatial navigation interventions in healthy older adults and analyze their efficacy.

**Methods:**

To identify spatial navigation interventions and assessments and investigate their effectiveness, four electronic databases were searched (Pubmed, Web of Science, CINAHL and EMBASE). Two independent reviewers conducted a screening of title, abstract and full-texts and performed a quality assessment. Studies were eligible if (1) published in English, (2) the full text was accessible, (3) at least one group of healthy older adults was included with (4) mean age of 65 years or older, (5) three or more spatial navigation-related training sessions were conducted and (6) at least one spatial ability outcome was reported.

**Results:**

Ten studies were included (*N* = 1,003, age-range 20–95 years, 51.5% female), only healthy older adults (*n* = 368, mean age ≥ 65) were assessed further. Studies differed in sample size (*n* = 22–401), type of training, total intervention duration (100 min–50 h), and intervention period (1–16 weeks).

**Conclusion:**

The spatial navigation abilities addressed and the measures applied to elicit intervention effects varied in quantity and methodology. Significant improvements were found for at least one spatial ability-related outcome in six of 10 interventions. Two interventions achieved a non-significant positive trend, another revealed no measurable post-training improvement, and one study did not report pre-post-differences. The results indicate that different types of spatial navigation interventions improve components of spatial abilities in healthy older adults. The existing body of research does not allow conclusions on transferability of the trained components on everyday life spatial navigation performance. Future research should focus on reproducing and extending the promising approaches of available evidence. From this, valuable insights on healthy aging could emerge.

**Trial Registration:**

This scoping review was preregistered at Open Science Framework (https://osf.io/m9ab6).

## Background

Many older adults perceive an autonomous and meaningful life within their community as a desirable goal and strive to live independently as long as possible (Portegijs et al., 2014). This is challenged by age-related processes leading to motor and cognitive degradation. One complex cognitive ability deteriorating with age is spatial navigation (e.g., planning to reach an intended destination and move through physical environments; Burns, 1999; Moffat et al., 2006; Iaria et al., 2009; Head and Isom, 2010). While the ability to navigate through familiar environments remains relatively stable, coping with unfamiliar environments decreases notably with increasing age (Colombo et al., 2017).

Whether older persons are able to live and function independently on a daily basis is therefore determined by finding their way around familiar and new environments, the ability to move outdoors, as well as adequate physical, cognitive, and mental health (Portegijs et al., 2014; Claessen et al., 2015; Muffato et al., 2020). The steady decline or even loss of any of these capabilities immediately affects the others leading to a vicious circle of fading independence. Decreasing navigational skills are further known to be an early predictor of pathological aging processes like dementia or mild cognitive impairment and are common in trauma-induced contexts (Moffat, 2009; Claessen et al., 2015; Boccia et al., 2019).

This decline can cause fear of not finding a particular destination like the way home, or even getting lost (Phillips et al., 2013; Saajanaho et al., 2015). To reach a destination in the environment, humans primarily use two different reference frames: the egocentric and the allocentric reference frame (Kitchin, 1994; Gramann et al., 2005). The egocentric reference frame refers to the body position from a first-person perspective and integrates landmarks along the way related to the current position and orientation. In comparison, the allocentric reference frame is rather a bird’s eye representation of the environment, possibly including one’s body position and orientation but not taking it as a central reference point (Gramann, 2013; Colombo et al., 2017). In this reference frame, external landmarks are collected to generate a mental representation of the environment, often called a “cognitive map” (Kitchin, 1994; Epstein et al., 2017). Studies in virtual environments showed that spatial abilities, which rely on the egocentric frame of reference, remain stable with age, while allocentric abilities deteriorate (Moffat et al., 2006; Iaria et al., 2009; Fricke and Bock, 2018). This, in turn, can hamper the physical activity and social participation behavior of older adults and might reflect on a person’s life-space mobility (i.e., the social and physical environment a person moves in on a daily basis), safety, and overall well-being (Burns, 1999; Taylor et al., 2019; Muffato et al., 2020).

Next, to the individuals’ cognitive and physical conditions, precursors of smaller life-spaces appear to be disadvantageous neighborhood properties, transportation issues, and lower levels of education and income (Lo et al., 2016). A larger life-space has been associated with a higher quality of life and good physical, cognitive, and psycho-social health (Baker et al., 2003; Sejunaite et al., 2017; Taylor et al., 2019). While the link between reduced life-space mobility and age-related decline of these properties has been well established, decreasing life-space also appears to be a predictor of nursing home admission (Sheppard et al., 2013) and mortality in older persons (Boyle et al., 2010; Mackey et al., 2014; Kennedy et al., 2017). An approach to counter these effects can be found in the evidence that both, the aging body and aging brain remain plastic, and capabilities can be improved through specific cognitive or motor training (Kramer et al., 2004; Erickson and Kramer, 2008; Park and Bischof, 2013; O’Callaghan et al., 2018). To link these three dimensions (environmental conditions, physical and cognitive abilities) and to help older adults maintain adequate resources for mobility, specific training interventions are required.

Numerous studies have investigated the training of spatial navigation abilities within pathologic populations to facilitate autonomy. A variety of different training strategies like augmented or virtual reality training, behavioral techniques, route and landmark learning, and combinations with motor-cognitive training approaches yielded very promising results (McGilton et al., 2003; Provencher et al., 2008; Sejunaite et al., 2017).

Despite these encouraging findings, little research has explored so far whether and how spatial navigation can be trained in a healthy older population. Closing this research gap is crucial, as effective training methods could impact significantly how healthy older adults keep up an independent life for as long as possible. This includes maintaining higher levels of physical and cognitive performance, living autonomously, remaining in familiar surroundings, having the possibility for social support and participation, having lower mental health incidences, and higher satisfaction with life, thereby taking a load off the health care system.

Thus, aiming to summarize previous and to facilitate future research in this field, this scoping review systematically reports the current knowledge about intervention studies addressing spatial navigation in healthy older adults. Furthermore, this scoping review provides the applied intervention characteristics and spatial ability-related outcome measures to address the following questions:

1. Which interventions for healthy older adults can be identified targeting spatial navigation abilities?
2. What spatial ability-related assessments were used in those studies to evaluate the effectiveness of the respective spatial interventions for healthy older adults?
3. Which effects of spatial navigation interventions for healthy older adults were observed on spatial abilities?

## Materials and methods

### Search strategy

**Figure 1:**
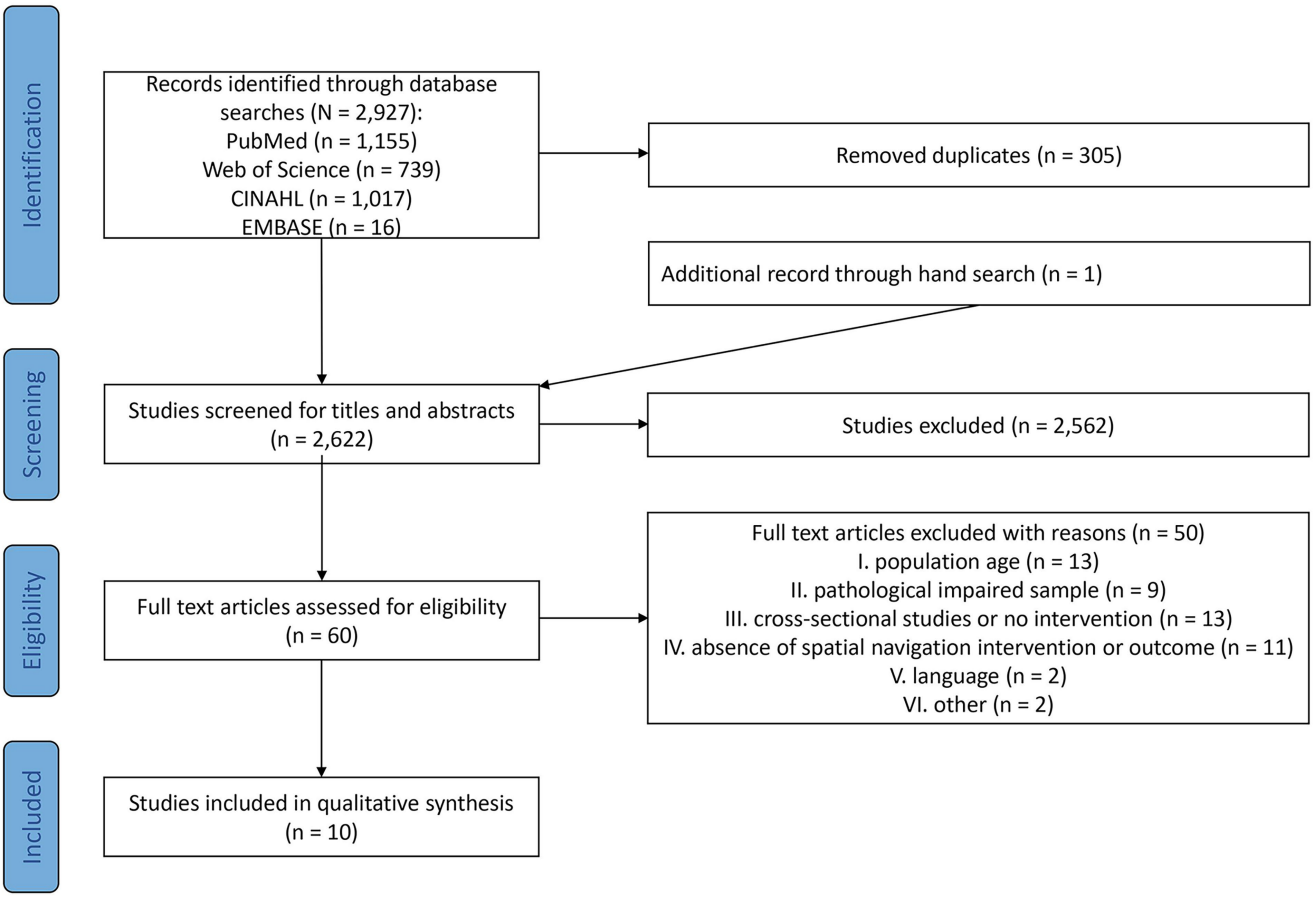
PRISMA flow diagram (according to Moher et al., 2011).

Relevant literature was identified by systematically searching the electronic databases Pubmed, Web of Science, CINAHL, and EMBASE. Systematic searches were conducted from the inception of the respective database up until September 2021. The search strategy was developed and refined according to the PICOS scheme (P = healthy older adults, I = training of spatial navigation, C = control groups, O = motor or cognitive or spatial ability outcomes, S = study design), advised by a librarian. All search results were exported into Mendeley (version 1803). After the removal of duplicates, the remaining citations were uploaded to Rayyan QCRI (Ouzzani et al., 2016), a free-of-charge web application that allows collaboration on systematic reviews. Two reviewers (MF, CM) independently screened the titles and abstracts of all results for eligibility according to the inclusion and exclusion criteria. In case the information provided in the title and abstract was insufficient to decide whether an article should be included or excluded, the full-text version of these articles was retrieved and screened for eligibility. Afterward, full-text versions of all potentially relevant studies were obtained and assessed for inclusion by both reviewers. Disagreements were resolved by discussion and consensus. The PRISMA Flow Diagram (Moher et al., 2011) shows the results of the study selection process and documents the reasons for study exclusion (Figure 1) and an example of the coherent search string for the final search on each database is depicted in Appendix 1. For PubMed and CINAHL the results were limited to humans by using the filter function. Grey literature was searched through Google Scholar. Next to that, reference lists of identified articles and review articles in the associated research field were hand-searched to detect additional relevant publications that were not identified by the databases.

### Data charting

Both reviewers documented their data charting process with a modified version of the ‘Data collection form for intervention reviews: RCTs and non-RCTs’. Applied items were key study characteristics like the author(s), year of publication, study design, the aim of the study, population characteristics, intervention characteristics, control interventions, follow-ups, drop-outs, assessed outcomes, and self-reported limitations.

Additionally, information was collected about the content of spatial navigation interventions, training modalities, spatial ability-related outcome measures and outcomes. It should be noted, that only behavioral spatial ability-related outcome measures are reported and discussed, disregarding additional reported data (e.g., imaging data). Data were listed as a mean and standard deviation (SD) if reported. To capture all relevant information, the form was refined in an iterative process and data were updated continuously. Extracted data of both researchers were compared and discrepancies were resolved by discussion. According to the research questions, data about study design, study aim, population and intervention characteristics, spatial ability-related outcome measures and outcomes were used to identify relevant studies and to summarize the applied spatial ability-related assessments and training effects on spatial navigation abilities.

### Inclusion and exclusion criteria

Included studies met the following criteria: (I) published in the English language, (II) accessible as a full-text version, (III) at least one group of healthy older adults included with a (IV) mean age of 65 years or older, (V) three or more spatial navigation training sessions conducted and (VI) at least one spatial ability-related outcome reported.

Studies were excluded if they were (I) only cross-sectional or (II) books, reviews, editorials, single case studies, conference abstracts, or interviews. A sample of solely (III) pathologically impaired participants also led to exclusion as well as (IV) the absence of spatial outcomes.

### Quality assessment

A quality assessment was performed according to Kmet et al. (2004). This critical appraisal was performed to depict the methodological quality of the included articles and to discriminate the meaningfulness of derived evidence. The original quality assessment contains 14 criteria, of which 11 were applicable to the included studies (*cf.* criteria 1–11 in Table 1). For each criterion, a maximum of two points was given by two independent reviewers, depending on the degree to which the criterion was met (“yes” = 2, “partial” = 1, “no” = 0, “x” = n/a). Discrepancies were resolved by discussion. For each article, a total score was summed up across relevant items and divided by the total possible score reduced by the number of not applicable criteria times two [i.e., if 10 criteria were fully met but one was not applicable, the score was 20/(22–1*2) = 1]. Due to the limited literature available on this topic, the quality assessment was not used as an inclusion criterion but as a measure of study quality. Referring to Curry et al. (2021) and Lannes et al. (2021) studies with a score of 0.80 and above were considered high quality, whereas scores between 0.70 and 0.79 were defined as good quality. Studies scoring between 0.50 and 0.69 were classified as moderate quality. Studies below the score of 0.50 were classified as poor.

**Table 1:** Quality assessment (according to Kmet et al. (2004)).

**Author**	**Publication year**	**Item 1**	**Item 2**	**Item 3**	**Item 4**	**Item 5**	**Item 6**	**Item 7**	**Item 8**	**Item 9**	**Item 10**	**Item 11**	**Total score/max. score**	Quality score [max. score (−n/a*2)]
Binder et al.	2016	2	2	2	2	1	2	1	2	2	2	2	20/22	0.90
Kober et al.	2013	2	2	1	2	n/a	2	1	2	2	2	2	18/20	0.90
Lövdén et al.	2012	2	2	2	2	1	2	1	2	2	2	2	20/22	0.90
Mitolo et al.	2016	2	2	1	2	1	2	1	1	2	2	2	18/22	0.81
Nemmi et al.	2016	2	2	0	2	0	1	1	1	2	2	2	15/22	0.68
Schaie et al.	1987	1	1	2	2	0	2	2	1	1	1	1	14/22	0.63
Serino et al.	2017	2	2	1	2	1	2	1	1	2	2	2	18/22	0.81
Whitlock et al.	2012	2	2	2	2	0	2	1	1	2	2	2	18/22	0.81
Wiener et al.	2012	2	2	1	2	n/a	1	1	1	2	2	2	16/20	0.80
Willis & Schaie	1986	1	1	2	2	0	2	2	1	1	1	1	14/22	0.63

2 = yes; 1 = partially, with general remarks; no = 0; n/a = not applicable: Note: Studies with a score above 0.80 were considered high quality, with scores between 0.70 and 0.79 were considered good quality, with scores between 0.50 and 0.69 were considered moderate quality, and with a score below 0.50 were considered poor quality.

Item 1: sufficient description of question/objective.

Item 2: appropriate study design.

Item 3: appropriate method of participant selection or source of information/input variables.

Item 4: sufficient description of patient characteristics.

Item 5: description available of interventional and random allocation (if possible).

Item 6: report of means of assessment with outcome measures well defined and robust to measurement/misclassification bias.

Item 7: appropriate sample size.

Item 8: appropriate analytic methods and method description.

Item 9: report available of some estimates of variances in main outcomes.

Item 10: sufficiently detailed report of the results.

Item 11: conclusions supported by the results.

## Results

### Study selection

As shown in Figure 1, the systematic search within the databases PubMed, Web of Science, CINAHL and EMBASE identified 2,927 articles for consideration. Three hundred-five duplicates were removed. After title and abstract screening, a total of 60 full-text articles were included for further examination. The main reasons for exclusion were for example participants, that did not meet the inclusion criteria for age, pathological impaired samples without a healthy control group, or the absence of spatial navigation interventions or spatial ability-related outcomes. Two of the studies were dropped due to publication in Italian. In the end, 10 articles were incorporated into the qualitative synthesis.

### Study characteristics

Table 2 gives a comprehensive overview of the general study characteristics of the included articles. Table 3 provides a deeper insight into the interventions performed, and their training modalities as well as the spatial ability-related assessments used. It further briefly presents the findings concerning the applied spatial ability-related measures.

**Table 2:** Characteristics of included spatial navigation interventions.

**Authors and year**	**Title**	**Study design**	**Study aims**	**Sample size, age and gender**	**Group allocation**	**Training type**
Binder et al., 2016	Multi-domain training enhances attentional control	Randomised controlled trial	Evaluation of the effects of three different single-domain and one multi-domain iPad-based interventions on attentional control	N = 84; IG1: n = 22, 70.50 ± 3.05, 14 f/8 m IG2: n = 21, 68.81 ± 2.48, 11 f/10 m **IG3: n = 20, 68.95 ± 2.76, 11 f/9 m** IG4: n = 21, 69.62 ± 2.85, 13 f/8 m 12 dropouts: 2 excluded due to low MMSE-scores, 10 excluded from analyses due to health	IG1: Inhibition training group IG2: Visuomotor function training group IG3: Spatial navigation training group IG4: Multi-domain training group	Individual training
Kober et al., 2013	Virtual reality in neurologic rehabilitation of spatial disorientation	Controlled clinical trial	Effects of VR-route-finding-training on spatial abilities in neurological patients with spatial disorientation and healthy older adults	N = 22; IG: n = 11, 66.09 ± 3.30, 6 f/5 m **CG: n = 11, 66.18 ± 2.97, 6 f/5 m**	IG1: VR-route finding-training CG: VR-route-finding-training	Individual training
Lövdén et al., 2012	Spatial navigation training protects the hippocampus against age-related changes during early and late adulthood	Randomised controlled trial	Benefits of VR-navigation training on spatial navigation and hippocampal volumes in younger and older men	N = 91; **IG1: n = 23, 65.3 ± 2.8, 0 f/23 m** IG2: n = 23, 25.1 ± 2.8, 0 f/23 m CG1: n = 24, 64.6 ± 2.9, 0 f/24 m CG2: n = 21, 27.01 ± 2.5, 0 f/21 m 27 dropouts: 12 excluded due to brain abnormalities, 6 due to imaging problems, 9 due to lack of motivation, health issues or personal reasons	IG1 & IG2 (HOA & HYA): spatial navigation training in virtual zoos CG1 &2 (HOA & HYA): Walking on a treadmill at a preferred pace	Individual training
Mitolo et al., 2017	How to enhance route learning and visuospatial working memory in aging: a training for residential care home residents	Randomised controlled trial	Efficacy of spatial navigation training on route-learning, sense of direction and spatial anxiety in healthy older nursing home residents	N = 30; **IG: n = 15, 85.80 ± 8.53, 12 f/3 m** CG: n = 15, 85.40 ± 4.99, 12f/3 m	IG1: route-learning training CG: non-spatial group activities (reading newspapers, etc.)	Group training (group size has not been described)
Nemmi et al., 2017	Does aging affect the formation of new topographical memories? Evidence from an extensive spatial training	Randomised controlled trial	Comparison of healthy older and healthy younger adults in route learning and survey learning	N = 39; **IG1: n = 16, 65.62 ± 5.13, 4 f/12 m** IG2: n = 23, 25.4 ± 1.08, 11 f/12 m 8 dropouts: missing data due to technical problems	IG1: training path A from the route perspective and path B from the survey perspective and vice versa IG2: training path A and B from vice versa perspectives	Individual training
Schaie et al., 1987	Effects of cognitive training on primary mental ability structure	Controlled clinical trial	Demonstration of training gain on the primary mental abilities inductive reasoning and spatial orientation	N = 401, 72.5 ± 6.41, 224 f/177 m **IG1: n = 118** IG2: n = 111 CG: n = 172	IG1: spatial orientation training IG2: inductive reasoning training CG: no treatment	Individual training
Serino et al., 2017	A novel virtual reality-based training protocol for the enhancement of the “mental frame syncing” in individuals with alzheimer’s disease: a development-of-concept trial	Randomised controlled trial	Evaluation of a VR-based training of syncing between allocentric viewpoint-dependent and allocentric viewpoint-independent representations in healthy older adults and older adults with AD	N = 28; IG1: n = 10, 86.60 ± 6.13, 9 f/1 m **IG2: n = 8, 86.62 ± 6.19, 4 f/4 m** CG: n = 10, 88.70 ± 3.59, 8 f/2 m	IG1 (AD): VR-training IG2 (HOA): VR-training CG (AD): traditional cognitive rehabilitation	Individual training
Whitlock et al., 2012	Individual differences in response to cognitive training: Using a multi-modal, attentionally demanding game-based intervention for older adults	Controlled clinical trial	Effects of a video game-based intervention in attention and spatial navigation in healthy older adults	N = 39; **IG: n = 19, 68.58 ± 4.38, 9f/10 m** CG: n = 20, 66.80 ± 4.93, 11 f/9 m	IG: video gaming CG: no treatment	Individual, unaccompanied training at home
Wiener et al., 2012	Route repetition and route retracing: effects of cognitive aging	Controlled clinical trial	Investigation of age-related differences in route repetition and route retracing	N = 40; IG1: n = 20, 20.53 ± 1.84, 8 f/12 m **IG2: n = 20, 69.45 ± 5.48, 11 f/ 9 m** 2 drop-outs (HOA): chance level was not reached in Route Direction Task	IG1 & 2 (HYA & HOA): VR-training	Individual training
Willis and Schaie, 1986	Training the elderly on the ability factors of spatial orientation and inductive reasoning	Controlled clinical trial	Examination of cognitive training effects in participants from a longitudinal research program	N = 229, 72.8 ± 6.41; **IG1: n = 118, 66 f/52 m** CG: n = 111, 66 f/45 m	IG: spatial orientation training CG: inductive reasoning training	Individual training at home, guided by one of two trainers

IG: intervention group; CG: control group; VR: virtual reality; VE: virtual environment; AD: Alzheimer’s Disease; HOA: healthy older adults; HYA: healthy younger adults; MMSE: Mini-Mental State Examination. The target sample (healthy older adults) was highlighted in bold.

**Table 3:** Spatial navigation intervention contents and outcomes.

**Authors and year**	**Description of the spatial navigation intervention**	**Training duration, no. of sessions and training period**	**Addressed spatial abilities**	**Spatial measures**	**Results on spatial measures**
Binder et al., 2016	iPad-based intervention of 5 mini-games (6–10 min. each) in fixed order, adaptive and progressive task difficulty, addressing (1) inhibition, (2) visuomotor function and (3) spatial navigation Spatial navigation training: 5 mini-games consisting of 2D-and 3D-VEs with crossroads tasks requiring memory and recall of paths	45–50 h, 5 × 45–60 min. per week for 10 weeks	visuospatial short-term memory mental rotation visual–spatial short-term memory	Corsi Block Forward Test (CBFT, subtest of Wechsler Memory Scale Revised, Härting et al., 2000) 3D Spatial Orientation Test (3D SOT, Vienna Test System, Bratfisch and Hagmann, 2012) City Map Path Learning Test (CMPLT, subtest of Berlin Intelligence Structure Test, Jäger et al., 1997)	Spatial navigation (composite scores of CBFT, 3D SOT, CMPLT) showed a statistical trend for improvement in IG3 & IG4
Kober et al., 2013	Verbally guided VR navigation training, divided into 2 parts: Part 1 (learning): passive navigation training in a virtual City (aerial and first-person view, 2x2m projection screen) Part 2 (retrieval): participants verbally command direction decisions at each intersection learning and retrieval repeated until correct max. 3 routes per session	100 min., 5 x *ca.* 20 min.	visual–spatial memory visual short-term memory capacity, visuospatial learning spatial perception visual perception route learning	Benton Visual Retention Test (Benton, 1990) Corsi Block-Tapping Test (CBTT, subtest of Vienna Test System) Spatial imagination Test (subtest of LPS 50+, Sturm et al., 1993) Visual Pursuit Test (LVT, Vienna Test System. Psychological assessment [Internet], 2022) VR route-finding performance (first and fifth training session) (weighted total score including the number of mistakes per route and the number of correctly learned routes)	The raw score increased significantly in healthy older CG. No significant differences in CBTT. Performance increased significantly for IG post-training Non-significant improvements in LVT total score (IG, CG); significant faster response times for LVT at post-tests (IG). Performance increased significantly after VR training in healthy older adults.
Lövdén et al., 2012	Search for animals and the exit in virtual zoos while walking at a comfortable speed on a treadmill (animals displayed on screen in random order; new zoo after 4 trials)	35 h, 2–3 × 50 min. per week for 16 weeks (total 42 sessions)	route learning & survey learning spatial orientation mental rotation ability spatial episodic memory spatial episodic memory spatial episodic memory visuospatial short-term working memory	Navigation performance in virtual zoos (number of animals and exits found) Magnetic resonance imaging Guilford-Zimmerman spatial orientation test (Guilford and Zimmerman, 1948) Mental Rotation Test (Vandenberg and Kuse, 1978) Route Memory Test (subtest of the Berlin Intelligence Structure (BIS), Jäger et al., 1997) Location Memory (subtest of the BIS, Jäger et al., 1997) Object-position Memory (Schmiedek et al., 2010) Spatial 2-back (Schmiedek et al., 2010)	Significant performance improvements in both intervention groups (OHA, YHA). Non-significant larger performance improvement in older adults. Stable hippocampal volumes during training (age-related decline displayed in CGs) Non-significant trend towards improvement (OHA, YHA), navigation groups improved more than walking groups. No significant differences found in Mental Rotation Test, Route Memory Test, Location Memory, Object-position Memory, Spatial 2-back.
Mitolo et al., 2017	Behavioural spatial navigation intervention, divided into 3 parts: Part 1 (sessions 1–3): attitudes to spatial navigation (raising awareness about navigation strategies and reflection) Part 2 (sessions 4–9): routes reproduction sessions (route learning and reproduction in the same and reverse direction inside the nursing home, theoretical map use training in hometown) Part 3 (sessions 10–14): routes mental representation (preparation of a group excursion using maps, conduction of group excursion in hometown and photographing landmarks, a reflection of the experienced route, and landmark sequence training)	14 h, 1–2 × 60 min. per week for 8 weeks	route learning visuospatial short-term memory visuospatial working memory tasks visuospatial working memory tasks visuospatial working memory tasks sense of direction, spatial representation and orientation strategies spatial representation strategies spatial abilities level of anxiety in spatial orientation performance confidence in spatial tasks	Route Learning Test (Mitolo et al., 2017; 3 subtests, (I) walking routes within a matrix of 25 squares on the floor, (II) watching the experimenter walking routes, (III) learning routes presented on a map) Forward Corsi Blocks Test (Corsi, 1972; Mammarella et al., 2008) Backward Corsi Block Test (Corsi, 1972; Mammarella et al., 2008) Pathway Span Task (PST, Mammarella et al., 2008) Jigsaw Puzzle Test (Borella et al., 2007) Sense of Direction and Spatial Representations Questionnaire (SDSRQ, revised by Pazzaglia et al., 2000) Attitude to Environmental Tasks Questionnaire (AETQ, adapted by Pazzaglia et al., 2004) Spatial Anxiety Scale (SAS, adapted by Lawton, 1994) Spatial Self-Efficacy Questionnaire (SSEQ, adapted by Pazzaglia et al., 2004)	Significant improvement in all 3 subtests in IG, also at 3-month follow-up; no improvement in CG. Non-significant gains at post-test and follow up between IG and CG, favoring IG Significant immediate and long-term gains in IG Significant immediate and long-term gains in IG No significant immediate, longterm or maintenance gains Significant gains at post-test and follow up between IG and CG, favoring IG No change was observed in individuals’ opinion about spatial items collected in SDSRQ, AETQ, SAS and SSEQ
Nemmi et al., 2017	Route and survey learning sessions, using screenshots of 46 crossroads of two pathways (path A and B; similar length) Session 1: path and survey perspective of both routes with immediate retrieval Session 2–4: intensive RL & SL: half path A in route, path B in survey perspective and vice versa Session 5: learning + retrieval tasks	5 training sessions on consecutive days, duration per session has not been described	route learning survey learning	Route Task (RT) Survey Task (ST)	Significant improvement in reaction times in healthy older adults (session 1 to 2, learning continued in ongoing sessions) significant improvement of correct answers in healthy older adults
Schaie et al., 1987	The majority of participants were trained at home, no further information about training content was described	5 h, 1–2 × 60 min. per week for 4 weeks	two-dimensional mental rotation ability	Primary Mental Abilities Space (Thurstone, 1948) Object Rotation (Schaie et al., 1987) Alphanumeric Rotation (Willis and Schaie, 1986)	There was no statistical evaluation of the intervention effects.
Serino et al., 2017	Training started with a short VR technology briefing and was then structured in 2 parts: Part 1: encoding phase (objects had to be found in a VE, the number of objects increased progressively) Part 2: retrieval phase (object locations had to be revisited, starting from another position)	200 min, approx. 3 × 20 min. per week for 3–4 weeks (total 10 sessions)	executive functions short-term memory short and long-term spatial memory	Frontal Assessment Battery (Appollonio et al., 2005) Digit Span Test (Monaco et al., 2013) Corsi Block Test (both versions, Corsi, 1972; Monaco et al., 2013)	General increase, but no significant improvements in HOA. No significant difference in pre-post comparison of HOA No significant difference in pre-post comparison of HOA
Whitlock et al., 2012	Home-based playing of “World of Warcraft,” including the navigation through the environment	14 h, 7 × 60 min. per week, for 2 weeks (120 min. Pre-training session for demonstration and practice)	mental rotation ability spatial orientation spatial visualization	Mental Rotation Test (Peters et al., 1995; Vandenberg and Kuse, 1978) Object Perspective Test (Kozhevnikov and Hegarty, 2001) Paper Folding Test (administered only at post-test; Ekstrom et al., 1976)	No significant changes in pre-post comparison. No significant differences were found for IG & CG.
Wiener et al., 2012	Each session was structured in 2 parts: Part 1 (training): Passive travel through a VR maze twice with unique landmarks on each of the 11 visited 4-armed intersections Part 2 (test): 18 trials with 3 subtasks each (balanced, randomized order for route repetition or route retracing condition in (1) Route direction task, (2) Intersection direction task, and (3) Landmark sequence task)	6 sessions including the training and test phase; training duration and frequency has not been described	route learning route learning route learning	Route direction task Intersection direction task Landmark sequence task	Significant improvements in route repetition condition for HOA and HYA, significant lower accuracy in HOA for route retracing condition significant differences analyzing direction, session 1–3 and 4–6 and age group, HOA did not improve in route retracing condition main effects of age and session, but not direction (forward and backward); performance of HOA and HYA improved over sessions
Willis and Schaie, 1986	i.e. development of concrete terms for various angles, practice with manual rotation of figures prior to mental rotation, practice with rotations of drawings of familiar objects prior to the introduction of abstract figures, subject-generated names for abstract figures, having subjects focus on two or more features of the figure during rotation	5 h, 5 × 60 min. within 2 weeks	two-dimensional mental rotation ability three-dimensional mental rotation ability	Primary Mental Abilities Space (Thurstone, 1948) Object Rotation (Schaie et al., 1987) Alphanumeric Rotation (Willis and Schaie, 1986) Cube Comparisons (Ekstrom et al., 1976)	spatial measurements were grouped as one factor; pre-post-test factor gain scores show significant improvement of IG1 in spatial orientation

AD: Alzheimer’s Disease; CG: control group; HOA: healthy older adults; HYA: healthy younger adults; IG: intervention group; MMSE: Mini-Mental State Examination; RL: route learning; SL: survey learning; VE: virtual environment; VR: virtual reality.

### Participant characteristics

Summarizing all records, a total of 1,003 adults participated in the studies (*cf.* Table 2), this includes younger or pathologically impaired comparison groups. Participants’ age ranged from 20 to 95 years, however, the older age group that was further assessed had a mean age of 65 years and above. Overall, 516 women and 487 men were included in the research activities. Three hundred-sixty-eight of these participants were healthy older adults receiving a spatial navigation-related intervention, while the others either had pathologic impairments or had been younger, healthy control participants. One study included men only (Lövdén et al., 2012) and two studies included considerably more female participants (Mitolo et al., 2017; Serino et al., 2017), the gender distribution in the remaining studies was approximately even.

### Quality assessment

Studies included in this systematic scoping review used heterogeneous designs and differed significantly in methodological quality (Table 1). Six studies were rated as high quality while one study was of good quality. Three studies were classified as moderate quality, none of the studies were considered of poor quality. It should be noted that the studies with the lowest scores omitted to provide sufficient and structured descriptions of their randomization processes, thereby reducing reproducibility. For two of them, this may be a concern of outdated reporting structures since they were published in the 1980s (Willis and Schaie, 1986; Schaie et al., 1987). In summary, the overall quality of the study pool is moderate to high.

### Intervention characteristics

The included studies (*N* = 10) differed in the interventions provided as well as the training methods (*cf.* Table 3). Whereas one study implemented a group intervention (Mitolo et al., 2017), all remaining studies performed one-on-one trainings. Of those, two studies trained their participants at home, one guided by a trainer (Willis and Schaie, 1986), the other one unaccompanied (Whitlock et al., 2012). All other studies provided training to their participants in a laboratory setting. In terms of training methods, one study implemented iPad-based mini-games (Binder et al., 2016), another study instructed participants to play an original computer game (Whitlock et al., 2012). In four studies participants performed active and passive movements in virtual environments (Lövdén et al., 2012; Wiener et al., 2012; Kober et al., 2013; Serino et al., 2017), one study conducted real-world behavioral training (Mitolo et al., 2017), one trained picture-based (Nemmi et al., 2017), and another study proceeded real-world spatial activities as well as cognitive spatial activities (Willis and Schaie, 1986). Schaie et al. (1987) did not provide detailed information about the type of training implemented. Moreover, training modalities differed across the studies, i.e., training periods ranged from one to 16 weeks with the number of sessions extending from five to 50 sessions in total, while the overall training duration varied from 100 min to 50 h with individual training sessions extending from 20 to 60 min. Two studies did not describe their training modalities in sufficient detail (Wiener et al., 2012; Nemmi et al., 2017). Finally, training frequency varied from one to seven sessions per week.

### Spatial ability-related outcome measures

All experimental designs applied at least one form of spatial outcome measure with most studies utilizing more than one spatial ability-related assessment (*cf.* Table 3). These assessments recorded (visuospatial) short-term memory, variations of the Corsi Block Test (Kober et al., 2013; Binder et al., 2016; Mitolo et al., 2017; Serino et al., 2017), the City Map Path Learning Test (Binder et al., 2016), Pathway Span Task, Jigsaw Puzzle Test (Mitolo et al., 2017), the Spatial 2-back (Lövdén et al., 2012) or the Digit Span Test (Serino et al., 2017). Visual perception and visual memory were rated with the Benton Visual Retention Test and the Visual Pursuit Test (Kober et al., 2013). Moreover, spatial perception and mental rotation abilities were examined utilizing the 3D Spatial Orientation Test (Binder et al., 2016), Primary Mental Abilities Space, Object Rotation (Willis and Schaie, 1986; Schaie et al., 1987), Alphanumeric Rotation and the Mental Rotation Test (Lövdén et al., 2012; Whitlock et al., 2012), the Spatial Imagination Test (Kober et al., 2013) or Cube Comparison (Willis and Schaie, 1986). Further, Lövdén et al. (2012) and Kober et al. (2013) assessed VR route-finding performance or navigation performance tasks to evaluate route learning and route knowledge, while Wiener et al. (2012) and Nemmi et al. (2017) implemented intervention-specific route and direction tasks. Alternatively, the Route Learning Test and the Landmark Sequence Test were used to measure these spatial outcomes. Spatial orientation was assessed by the Object Perspective Tests (Whitlock et al., 2012) and the Guilford-Zimmerman Test (Lövdén et al., 2012). The Route Memory Test, the Location Memory Test and Object Position Memory were applied to test episodic memory (Lövdén et al., 2012). Moreover, executive function and spatial visualization were assessed using the Frontal Assessment Battery (Serino et al., 2017) and the Paper Folding Test (Whitlock et al., 2012), respectively. Mitolo et al. (2017) further applied various spatial questionnaires like the Sense of Direction and Spatial Representations Questionnaire, the Attitude to Environmental Tasks Questionnaire, the Spatial Anxiety Scale, and the Spatial Self-Efficacy Questionnaire. It should be noted that the majority of the reported assessments only partially depict spatial aspects of the complex cognitive process of spatial navigation. Therefore, they might reflect spatial abilities that contribute to spatial navigation to a certain degree, but are not sufficient to map this complex cognitive ability in its entirety.

### Effects of spatial navigation training on spatial abilities

#### Visuospatial short-term/working memory

Five studies evaluated visuospatial short-term or working memory (Lövdén et al., 2012; Kober et al., 2013; Binder et al., 2016; Mitolo et al., 2017; Serino et al., 2017). After a specific route-learning training, significant between-group differences were found only for the backward version of the Corsi Block Test. Improvements were found in favor of the intervention group subsequent to the training phase and the follow-up-testing (Mitolo et al., 2017). A virtual route-finding training (Kober et al., 2013) and a virtual object-relocation training (Serino et al., 2017) did not lead to significant Corsi Block Test improvements in healthy older adults. Furthermore, no significant changes were found in the Digit Span Test (Serino et al., 2017), Jigsaw Puzzle Test (Mitolo et al., 2017) or the Spatial 2-back Test (Lövdén et al., 2012). The Pathway Span Test, however, yielded significant immediate and long-term gains in the intervention group (Mitolo et al., 2017). Binder et al. (2016) reported a composite score of the Corsi Block Forward Test, 3D-spatial orientation Test and the City Map Path Learning Test. They found a statistical trend for improvement in their spatial training group and their multi-domain training group, however, not in the groups performing inhibition or visuomotor function training. The raw score of the Benton Visual Retention Test improved significantly from pre-to post-test in healthy older adults, however, for the Visual Pursuit Test significant improvements emerged in response times, but not for the total score (Kober et al., 2013).

#### Mental rotation

Seven different tests for mental rotation and spatial perception abilities were performed in five studies. Binder et al. (2016) used the 3D Spatial Orientation Test as part of a composite score regarding spatial abilities. A positive trend was found for healthy older adults within the spatial and the multi-domain training group. The Primary Mental Abilities Space, the Object Rotation and the Alphanumeric Rotation Tests were used by Willis and Schaie (1986) and Schaie et al. (1987). While Schaie et al. (1987) did not perform a statistical evaluation of the intervention effects, Willis and Schaie (1986) added a Cube Comparison Test and found significant improvements in a composite score of these tests for older adults performing spatial navigation training. Whitlock et al. (2012) performed the Mental Rotation Test and found no significant changes for their video gaming intervention group or the passive control group, the same applies for the route and survey learning conducted by Lövdén et al. (2012). On the other hand, Kober et al. (2013) found significant improvements in spatial perception, conducting the Spatial Imagination Test.

#### Route and survey learning

Route learning was assessed in five studies. Kober et al. (2013) found significant route-finding improvements after their VR-based training. Significant improvements were also found for each of the three route-finding subtests used by Mitolo et al. (2017) after performing a behavioral spatial navigation-related intervention. Another study (Nemmi et al., 2017) used screenshots of crossroads to assess route learning. When participants had to decide on the following direction at each shown crossroad, reaction times improved significantly between training sessions one and two.

In a route direction task, both older and younger adults improved significantly comparing the first and the second half of the experiment (Wiener et al., 2012). In the same study, a route repetition subtask was conducted, the performance of the healthy older adults increased significantly. When participants were asked to indicate the direction required to return to the start location of the same route (route retracing), performance did not improve over time (Wiener et al., 2012). Performance differences were observed for the subsequent landmark sequence task, differentiating between the first half and the second half of the trials. Younger and older healthy adults improved significantly in this task. Survey learning as a spatial ability was addressed only once (Nemmi et al., 2017). Older adults significantly improved their performance in a pointing task, concerning the starting or ending point of a previously learned route. Lövdén et al. (2012) performed a task, which required combined route and survey learning. The old-aged intervention group performed significantly better after the training in a virtual zoo as well as compared to their age-matched walking control group.

#### Additional assessments

While Serino et al. (2017) found non-significant improvements regarding executive functions, measured by the Frontal Assessment Battery, Whitlock et al. (2012) did not find significant differences in spatial orientation and spatial visualization, measured by the Object Perspective Task and the Paper Folding Test, respectively. However, Lövdén et al. (2012) reported a non-significant trend towards improvement for spatial orientation tested by the Guilford-Zimmerman Spatial Orientation Test. Spatial episodic memory tested by Route Memory Test, Location Memory and Object-Position Memory did not improve after route and survey learning in a virtual zoo (Lövdén et al., 2012).

#### Questionnaires

None of the questionnaires regarding spatial orientation topics (e.g., sense of direction, spatial representation strategies, spatial anxiety and performance confidence in spatial tasks) showed changes from pre-to posttest.

## Discussion

The aim of this scoping review was to outline the current body of evidence on spatial navigation interventions for healthy older adults to increase spatial abilities. In addition, the intervention characteristics, applied spatial ability-related outcome measures, and results were summarized to describe relevant training effects of spatial ability training in healthy older adults. A scoping review of the literature was selected as approach to map out a wide-ranging picture of the available research in this field while still maintaining methodological rigor and diminishing bias (Munn et al., 2018).

### Summary of findings

A total number of 10 studies met the applied inclusion criteria and were subjected to a quality assessment. The quality of the included studies was moderate to high. Trials included a total number of 1,003 participants, 368 of those being healthy older adults and therefore target group of this scoping review. Overall, the study outcomes indicate that spatial abilities can be improved in healthy older adults by means of the designed training interventions. Six of nine studies reporting pre-post data found significant improvements for at least one of their spatial ability-related outcomes (Willis and Schaie, 1986; Lövdén et al., 2012; Wiener et al., 2012; Kober et al., 2013; Mitolo et al., 2017; Nemmi et al., 2017), however, applying a methodological variety of training protocols in terms of type of intervention, training periods and training durations. Aiming to identify the most relevant approaches to achieve spatial ability improvements in healthy older adults, several research gaps in the current literature were identified. While the available research is of moderate to high quality, the total number of published studies is low. Nevertheless, there is need for additional methodologically solid research including larger sample sizes, addressing specifically healthy older adults.

### Characteristics of observed spatial navigation training interventions

The first aim of this scoping review was to summarize the characteristics of existing spatial navigation interventions to enhance spatial abilities in healthy older adults. A total number of 10 different interventions could be identified. Overall, the implemented training methods varied widely, active and passive movement approaches in virtual environments (Lövdén et al., 2012; Wiener et al., 2012; Kober et al., 2013; Serino et al., 2017) were used most frequently, followed by gamification approaches (Whitlock et al., 2012; Binder et al., 2016), as well as picture-based, cognitive, and real world-approaches (Willis and Schaie, 1986; Mitolo et al., 2017; Nemmi et al., 2017). A recent meta-analysis of combined cognitive-motor interventions revealed positive effects on global cognition in older adults (Wollesen et al., 2020). Despite these promising findings only two of the studies included in this review applied this dual-task type of training protocol (Lövdén et al., 2012; Mitolo et al., 2017) and improved spatial ability-related outcomes successfully. However, the extent of the motor components concurrent to the cognitive training was not stated clearly by Mitolo et al. (2017).

This variety continued for the applied training periods (1–16 weeks), the overall training duration (100 min–50 h) and the duration of training sessions (20–60 min).

From an exercise science point of view, the effect of cognitive-motor interventions is highly dependent on the training control and the conduction of the training load (e.g., progression, intensity; Herold et al., 2019; Wollesen et al., 2020). Improvements in cognitive-motor performance were shown following a minimum overall duration of 330 min of progressive training (Wollesen and Voelcker-Rehage, 2014). This duration was reached in two of the included studies (Lövdén et al., 2012; Mitolo et al., 2017), both showing improvements in spatial ability-related outcomes. In contrast, Kober et al. (2013) found significant improvements in implementing cognitive training in virtual reality with a total training duration of 100 min.

### Characteristics of observed spatial ability-related outcome measures

The second aim was to give an overview of spatial ability-related measures to evaluate spatial navigation intervention effects in healthy older adults.

It became evident that the applied measures varied widely from established standardized tests (e.g., variations of the Corsi Block and Mental Rotation Test) addressing cognitive functions (e.g., visual–spatial short-term memory, mental rotation) to task-specific tests (e.g., route learning, survey learning) and spatial ability-related questionnaires. Six of the included studies applied standardized tests (Willis and Schaie, 1986; Schaie et al., 1987; Whitlock et al., 2012; Binder et al., 2016; Mitolo et al., 2017; Serino et al., 2017), while two of them used task-specific assessments (Wiener et al., 2012; Nemmi et al., 2017). It has to be assumed that spatial navigation is a complex multisensory process (Wolbers and Hegarty, 2010). However, a clear definition of spatial navigation and related cognitive processes is missing within the included studies. This hampers the attempt to link spatial cognitive tests with spatial navigation abilities.

Kober et al. (2013) as well as Lövdén et al. (2012) utilized a combination of standardized and task-specific tests. When evaluated by task-specific measurements (Wiener et al., 2012) or a combination of task-specific and standardized measurements (Lövdén et al., 2012; Kober et al., 2013), VR-based interventions led to significant improvements in at least one outcome measure. In contrast, when evaluated by standardized tests only, the VR-intervention failed to show significant improvements regarding spatial navigation abilities (Serino et al., 2017). Two gamified interventions made use of established outcome measures (Whitlock et al., 2012; Binder et al., 2016), both failing to reveal significant improvements. However, Binder and colleagues reported statistical trends of improvement for all their outcome measures. This might be due to the fact that Binder et al. (2016) developed iPad-based mini-games to specifically improve cognitive functions, while Whitlock et al. (2012) used a pre-existing computer game which had been developed without this purpose (World of Warcraft).

The studies performing cognitive training interventions using realistic scenarios (Nemmi et al., 2017: screenshots of existing crossroads; Mitolo et al., 2017: living areas in nursing homes), used either standardized measures (e.g., Forward Corsi Block Test; Mitolo et al., 2017) or task-specific measures (Route Task & Survey Task; Nemmi et al., 2017), both finding significant improvements in at least some of their spatial ability-related outcomes. In the study of Willis and Schaie (1986), a conceptual cognitive training and established assessments were conducted, and significant improvements were observed.

This overview depicts a broad variety of applied spatial ability-related outcome measures in relation to the different intervention approaches. The applied assessments depict partial aspects of the complex cognitive ability called spatial navigation, which needs to be considered when interpreting outcomes in terms of intervention effects. The majority of assessments capture small-scale spatial abilities only, which mainly cover two-and three-dimensional spatial visualization and spatial relations. Few of the used assessments survey large-scale spatial abilities, which refer to the concrete ability to orientate and navigate in complex large-scale environments (Yuan et al., 2019).

This does not allow for drawing conclusions on the eligibility of selected spatial ability-related outcome measures. Overall, it can be assumed that lab-based, standardized assessments of spatial abilities are suitable tools in basic research as they can depict specific cognitive aspects. At the same time, task-specific large-scale assessments might be more suitable to observe intervention effects since they illustrate the complex aspects of spatial navigation performance rather than underlying cognitive mechanisms. Furthermore, these tools could also be considered to reflect the transfer of the measured spatial ability to everyday spatial navigation more accurately.

### Effectiveness of observed spatial navigation training interventions

The third study aim was to identify effective spatial navigation training interventions for healthy older adults. Effect sizes generally provide statistical evidence for comparing the range of intervention effects across studies. Six of nine studies reported statistically significant improvements in at least one of the spatial ability-related outcomes post training. Only one of these studies (Mitolo et al., 2017) reported effect sizes reflecting training gains for spatial measures in healthy older adults. Mitolo et al. (2017) calculated effect sizes using Cohen’s *d* and found a range from 0.1 up to 1.3, reflecting small to large intervention effects for the spatial measures (Cohen, 1992), which significantly improved after training. Some of the other studies also examined effect sizes comparing intervention and control group, but not pre-and posttest data for each group. Since no comparable training effects can be derived from these data, future studies should report the related effect sizes to enable a cross-study evaluation of training effects.

To classify the relevance of specific training approaches, it is also important to evaluate their transfer effects on everyday functioning (Weng et al., 2019). Transfer effects can be divided in near vs. far transfer effects (post-training improvements of the trained tasks vs. improvements in cognitive function and untrained cognitive tasks or performance in everyday situations, respectively, Barnett and Ceci, 2002). Improvements were detected for both forms of interventions addressing cognitive functions like fluid intelligence (Baltes et al., 1989), working memory (Strobach and Huestegge, 2017), or attention (Cassavaugh and Kramer, 2009). Two of the included studies generated near transfer outcomes (Wiener et al., 2012; Nemmi et al., 2017) conducting task-specific assessments (e.g., route learning in a virtual environment), while four of them collected rather far transfer outcomes referring to cognitive functions by standardized measurements (Whitlock et al., 2012; Binder et al., 2016; Mitolo et al., 2017; Serino et al., 2017), but not everyday life spatial navigation performance. Furthermore, Lövdén et al. (2012) and Kober et al. (2013) evaluated both near and far transfer effects, although they also did not cover the everyday life spatial navigation performance. For the studies performed by Schaie et al. (1987) as well as Willis and Schaie (1986) an assignment of transfer effects was inconclusive, as the intervention contents were not clearly described. Overall, none of the included studies applied assessments examining everyday spatial navigation performance. This renders the evaluation of intervention effects in terms of spatial navigation improvements in everyday life impossible.

Nonetheless, the encouraging findings of this scoping review are particularly valuable when assuming an association of efficient spatial abilities and life space-mobility. Since the constriction of life space increases the risk of frailty (Xue et al., 2008), nursing home admission (Sheppard et al., 2013), cognitive decline (Crowe et al., 2008) and social isolation (Taylor et al., 2019), it seems all the more sensible to counteract such life space reduction by promoting and maintaining spatial navigation abilities. Whether these findings are transferable to other population groups needs to be investigated.

### Strengths and limitations

The main strength of this review is its structured and thorough methodological approach in line with the PRISMA Extension for Scoping Reviews (PRISMA-ScR; Tricco et al., 2018). As the literature search was performed in a structured, and reproducible manner, it can be assumed that nearly all relevant literature in this underexplored research field has been identified. This allows a comprehensive overview of the available evidence as well as the accentuation of gaps in knowledge. In addition, a quality assessment of the included studies was performed to classify their methodological quality as well as the significance of derived evidence.

Despite conducting a thorough search of the literature, only 10 studies met our inclusion criteria. This may be due to a variety of limiting factors. Even though the search strategy was inclusive, it cannot be ruled out that our choice of keywords and search strings impeded the detection of available literature. Further, only articles published in English were included, which might have led to the exclusion of additional relevant evidence in this emerging field of research. It appears that only little research has investigated this topic for this specific population group applying a structured study design including a pre-and posttest as well as at least three spatial navigation-related intervention sessions. Altogether, sample sizes of healthy older adults involved in the included studies were relatively low as this target group frequently served as control-or comparison group for individuals with pathological impairments or different age groups. Overall, the 10 included studies presented with a strong heterogeneity regarding their study designs, population characteristics, interventions, training modalities and outcome measures. This significantly hampered comparability of their findings. In line with the scope of this review, results were thus presented in a descriptive manner. Moreover, there is a need for diagnostically informative, comparable outcome measures with high reliability and validity that convey a meaningful picture of intervention effects.

## Conclusion

The ability to navigate successfully through known and unknown environments determines health-relevant aspects in old age. It ascertains spatial range of motion, which in turn interacts with substantial skills promoting an independent and satisfying lifestyle. Even though the characteristics of spatial navigation interventions appear to be well established in pathological contexts, little is known about how to prevent deterioration of spatial abilities in healthy older adults.

Concerning the research questions, this systematic scoping review reveals a promising contribution of effective but methodologically diverse interventions, implementing training approaches, like cognitive and cognitive-motor trainings in virtual environments, computer games, picture-based and real-world scenarios. This diversity is also reflected in the assessments applied, like established, standardized tests, task-specific assessments, or spatial navigation-related questionnaires. This diversity in the methods limits the comparability of the resultant training effects. Thus, methodologically comparable studies should be conducted to create explicit recommendations for successful spatial navigation training in healthy older adults. Nevertheless, six of nine studies reported statistically significant improvements in at least one of the spatial ability-related outcomes post-training as well as far transfer effects.

Since brain plasticity has been proven for other complex cognitive abilities (e.g., executive functions) in healthy older adults, future research into specific spatial navigation interventions might be a valuable contribution to health prevention and thus not only improve the independence and quality of life in older persons but also take some of the load off the health care systems.

Overall, this scoping review revealed open aspects that should be addressed in future training studies. These are (a) studies have to provide a clear definition of spatial navigation and the involved cognitive processes that are addressed in the training, (b) future studies should report effect sizes for the effect of the training intervention to enable a cross-study evaluation (c) future studies should address the positive benefits of the specific training regimes on near and far transfer of underlying spatial cognitive abilities and (d) future studies should investigate the transfer of positive effects of training to daily activities and real-life navigation.

Future research should focus on reproducing and extending the available approaches. This could for example imply approaches like the comparison of different types of interventions (e.g., VR and real-life approaches) to identify the most promising training approaches. Additionally, the comparison of different training modalities could be beneficial to reveal efficient ways to yield improvements following established training principles. The assessment of life space-mobility may be a valuable addition to depict far transfer achievements of spatial ability training in everyday life.

## Author contributions

MF and CM wrote the first draft of the manuscript. MF, CM, AW, C-PJ, TM, KG, and BW have been involved in the drafting and contributed significantly to the revision of this manuscript. All authors contributed to the article and approved the submitted version.

## Funding

We acknowledge support by the German Research Foundation and the Open Access Publication Fund of TU Berlin.

## Conflict of interest

The authors declare that the research was conducted in the absence of any commercial or financial relationships that could be construed as a potential conflict of interest.

## Publisher’s note

All claims expressed in this article are solely those of the authors and do not necessarily represent those of their affiliated organizations, or those of the publisher, the editors and the reviewers. Any product that may be evaluated in this article, or claim that may be made by its manufacturer, is not guaranteed or endorsed by the publisher.

## Abbreviations:

AD: Alzheimer’s disease
CG: control group
CT: controlled trial
IG: intervention group
HOA: healthy older adults
HYA: healthy younger adults
MMSE: Mini Mental State Examination
RCT: randomized controlled trial
RL: Route learning
SL: Survey learning
VR: virtual reality
VE: virtual environment.
