# Supplementary material for: Successful wayfinding in age: A scoping review on spatial navigation training in healthy older adults
Source: Front Psychol. 2022 Aug 16;13:867987. doi: 10.3389/fpsyg.2022.867987 (PMC9424919; doi:10.3389/fpsyg.2022.867987)
Supplement: Supplementary file 1 [file Data_Sheet_1.DOCX]

**Appendix 1: Coherent search strings for the final searches on each database**

**Web of Science**

1. ALL FIELDS: (spatial navigation) OR ALL FIELDS: (spatial orientation) OR ALL FIELDS: (spatial behavior) OR ALL FIELDS: (cue*) OR ALL FIELDS: (wayfinding) OR ALL FIELDS: (cognitive map*) OR ALL FIELDS: (allocentric) OR ALL FIELDS: (egocentric) OR ALL FIELDS: (route learning)
2. ALL FIELDS: (intervention*) OR ALL FIELDS: (exercise*) OR ALL FIELDS: (exercise therapy*) OR ALL FIELDS: (treatment*) OR ALL FIELDS: (training*)
3. ALL FIELDS: (age*) OR ALL FIELDS: (nursing home*) OR ALL FIELDS: (home* for the aged) OR ALL FIELDS: (nursing home resident*) OR ALL FIELDS: (senior citizen*) OR ALL FIELDS: (residential care) OR ALL FIELDS: (healthy older adult*) OR ALL FIELDS: (elderly)
4. ALL FIELDS: (postural balance) OR ALL FIELDS: (activity* of daily living) OR ALL FIELDS: (executive function*) OR ALL FIELDS: (accidental fall*) OR ALL FIELDS: (life space mobility) OR ALL FIELDS: (spatial navigation ability*) OR ALL FIELDS: (spatial orientation ability*)
5. #4 AND #3 AND #2 AND #1

**Pubmed**

1. ((((((((((((((spatial navigation[MeSH Terms]) OR (spatial orientation[MeSH Terms])) OR (spatial behavior[MeSH Terms])) OR (cues[MeSH Terms])) OR (spatial navigation)) OR (spatial orientation)) OR (spatial behavior)) OR (cues)) OR (wayfinding)) OR (cognitive map)) OR (allocentric)) OR (egocentric)) OR (route learning))
2. (((((((intervention[MeSH Terms]) OR (exercise[MeSH Terms])) OR (exercise therapy[MeSH Terms])) OR (intervention)) OR (exercise)) OR (exercise therapy)) OR (treatment)) OR (training)
3. ((((((((((aged[MeSH Terms]) OR (nursing homes[MeSH Terms])) OR (homes for the aged[MeSH Terms])) OR (aged)) OR (nursing homes)) OR (homes for the aged)) OR (nursing home residents)) OR (senior citizens)) OR (residential care)) OR (healthy older adults)) OR (elderly)
4. (((((((((((postural balance[MeSH Terms]) OR (activities of daily living[MeSH Terms])) OR (executive function[MeSH Terms])) OR (accidental falls[MeSH Terms])) OR (postural balance)) OR (activities of daily living)) OR (executive function)) OR (accidental falls)) OR (life space mobility)) OR (spatial navigation ability)) OR (spatial orientation ability))
5. (((((((((((((((((spatial navigation[MeSH Terms]) OR (spatial orientation[MeSH Terms])) OR (spatial behavior[MeSH Terms])) OR (cues[MeSH Terms])) OR (spatial navigation)) OR (spatial orientation)) OR (spatial behavior)) OR (cues)) OR (wayfinding)) OR (cognitive map)) OR (allocentric)) OR (egocentric)) OR (route learning))) AND ((((((((intervention[MeSH Terms]) OR (exercise[MeSH Terms])) OR (exercise therapy[MeSH Terms])) OR (intervention)) OR (exercise)) OR (exercise therapy)) OR (treatment)) OR (training))) AND (((((((((((aged[MeSH Terms]) OR (nursing homes[MeSH Terms])) OR (homes for the aged[MeSH Terms])) OR (aged)) OR (nursing homes)) OR (homes for the aged)) OR (nursing home residents)) OR (senior citizens)) OR (residential care)) OR (healthy older adults)) OR (elderly))) AND ((((((((((((postural balance[MeSH Terms]) OR (activities of daily living[MeSH Terms])) OR (executive function[MeSH Terms])) OR (accidental falls[MeSH Terms])) OR (postural balance)) OR (activities of daily living)) OR (executive function)) OR (accidental falls)) OR (life space mobility)) OR (spatial navigation ability)) OR (spatial orientation ability)))
6. (((((((((((((((((spatial navigation[MeSH Terms]) OR (spatial orientation[MeSH Terms])) OR (spatial behavior[MeSH Terms])) OR (cues[MeSH Terms])) OR (spatial navigation)) OR (spatial orientation)) OR (spatial behavior)) OR (cues)) OR (wayfinding)) OR (cognitive map)) OR (allocentric)) OR (egocentric)) OR (route learning))) AND ((((((((intervention[MeSH Terms]) OR (exercise[MeSH Terms])) OR (exercise therapy[MeSH Terms])) OR (intervention)) OR (exercise)) OR (exercise therapy)) OR (treatment)) OR (training))) AND (((((((((((aged[MeSH Terms]) OR (nursing homes[MeSH Terms])) OR (homes for the aged[MeSH Terms])) OR (aged)) OR (nursing homes)) OR (homes for the aged)) OR (nursing home residents)) OR (senior citizens)) OR (residential care)) OR (healthy older adults)) OR (elderly))) AND ((((((((((((postural balance[MeSH Terms]) OR (activities of daily living[MeSH Terms])) OR (executive function[MeSH Terms])) OR (accidental falls[MeSH Terms])) OR (postural balance)) OR (activities of daily living)) OR (executive function)) OR (accidental falls)) OR (life space mobility)) OR (spatial navigation ability)) OR (spatial orientation ability)))

**Embase**

1. (spatial AND ('navigation'/exp OR navigation) OR 'spatial orientation'/exp OR 'spatial orientation' OR 'spatial behavior'/exp OR 'spatial behavior' OR 'association'/exp OR 'association' OR wayfinding OR 'cognitive map'/exp OR 'cognitive map' OR allocentric OR egocentric OR route) AND ('learning'/exp OR learning)
2. ('intervention'/exp OR intervention OR 'exercise'/exp OR exercise) AND ('therapy'/exp OR therapy) OR 'treatment'/exp OR treatment OR 'training'/exp OR training
3. (((((('aged'/exp OR aged OR 'nursing'/exp OR nursing) AND homes OR homes) AND for AND the AND ('aged'/exp OR aged) OR 'nursing'/exp OR nursing) AND ('home'/exp OR home) AND residents OR senior) AND citizens OR residential) AND ('care'/exp OR care) OR healthy) AND older AND ('adults'/exp OR adults) OR 'elderly'/exp OR elderly
4. (((postural AND ('balance'/exp OR balance) OR 'daily life activity'/exp OR 'daily life activity' OR 'executive function'/exp OR 'executive function' OR accidental) AND ('falls'/exp OR falls) OR 'life space mobility'/exp OR 'life space mobility' OR spatial) AND ('navigation'/exp OR navigation) AND ('ability'/exp OR ability) OR spatial) AND ('orientation'/exp OR orientation) AND ('ability'/exp OR ability)
5. #1 AND #2 AND #3 AND #4

**CINAHL**

1. "spatial navigation*"
2. "spatial orientation*"
3. (MM "Spatial Behavior") OR "Spatial Behavior"
4. (MH "Cues") OR "Cue*"
5. "wayfinding"
6. (MM "Concept Mapping") OR "cognitive map*"
7. "allocentric*"
8. "egocentric*"
9. "route learning"
10. (S1 OR S2 OR S3 OR S4 OR S5 OR S6 OR S8 OR S9)
11. "intervention*"
12. (MM "Exercise+") OR "Exercise*"
13. (MM "Therapeutic Exercise+") OR "Exercise Therap*"
14. "treatment*"
15. "training*"
16. (S11 OR S12 OR S13 OR S14 OR S15)
17. (MM "Nursing Homes+") OR "Nursing Home*"
18. (MM "Aged+") OR "Age*"
19. (MH "Nursing Home Patients") OR "Nursing Home Resident*"
20. "home* for the aged" OR (MH "Housing for the Elderly")
21. "senior citizen*"
22. (MH "Residential Care") OR "Residential Care"
23. "healthy old* adult*"
24. S17 OR S18 OR S19 OR S20 OR S21 OR S22 OR S23
25. (MH "Balance, Postural") OR "Postural Balance" OR "Balance"
26. (MM "Activities of Daily Living+") OR "Activit* of Daily Living"
27. (MH "Executive Function") OR "Executive Function*"
28. (MH "Accidental Falls") OR "Accidental Fall*" OR "Fall*"
29. "life space mobilit*"
30. "spatial navigation abilit*"
31. "spatial orientation abilit*"
32. (S25 OR S26 OR S27 OR S28 OR S29 OR S30 OR S31)
33. S10 AND S16 AND S24 AND S32
34. S10 AND S16 AND S24 AND S32
